# Bioactive Peptide F2d Isolated from Rice Residue Exerts Antioxidant Effects via Nrf2 Signaling Pathway

**DOI:** 10.1155/2021/2637577

**Published:** 2021-09-29

**Authors:** Jinliang Liu, Qiang Wu, Tao Yang, Feiyan Yang, Tianyi Guo, Yaping Zhou, Shuai Han, Yi Luo, Ting Guo, Feijun Luo, Qinlu Lin

**Affiliations:** ^1^Hunan Key Laboratory of Grain-Oil Deep Process and Quality Control, Hunan Key Laboratory of Processed Food for Special Medical Purpose, Hunan Key Laboratory of Forestry Edible Resources Safety and Processing, National Engineering Laboratory for Deep Process of Rice and Byproducts, Central South University of Forestry and Technology, Changsha, Hunan 41004, China; ^2^Department of Clinic Medicine, Xiangya School of Medicine, Central South University, Changsha 410008, China

## Abstract

Studies have shown that the peroxidation caused by oxygen free radicals is an important reason of vascular endothelial dysfunction and multiple diseases. In this study, active peptides (F2ds) were isolated from the fermentation product of rice dregs and its antioxidant effects were approved. Human umbilical vein endothelial cells (HUVECs) stimulated by H_2_O_2_ were used to evaluate the antioxidation effect and its molecular mechanism in the oxidative stress model. F2d protected H_2_O_2_-induced damage in HUVECs in a dosage-dependent manner. F2d can reduce the expression of Keap1, promote the expression of Nrf2, and activate the downstream target HO-1, NQO1, etc. It means F2d can modulate the Nrf2 signaling pathway. Using Nrf2 inhibitor ML385 to block the Nrf2 activation, the protective function of F2d is partially lost in the damage model. Our results indicated that F2d isolated from rice exerts antioxidant effects via the Nrf2 signaling pathway in H_2_O_2_-induced damage, and the work will benefit to develop functional foods.

## 1. Introduction

Rice residue is a by-product of rice processing and its protein content is up to 60%. Rice dreg proteins are rich in methionine and cysteine, which are different from other plant or animal proteins [1], so rice residue is considered a source of high-quality protein [2]. Studies showed that many active peptides derived from rice have anti-inflammatory [3], lipid-lowering [4], antiageing [5], antioxidant [6], and blood pressure-lowering [7] effects. Although rice residue has so many advantages, a large amount of rice residue is used as animal feed or directly discarded and has not been fully exploited and utilized. Therefore, microbial fermentation of rice residues to obtain active peptides with certain physiological functions can provide an important basis for better utilization of rice residues and effectively alleviate the waste of rice residues. It has important significance in increasing the adding value of by-products of rice processing and promotes the sustainable development of the rice industry.

Reactive oxygen species (ROS) are produced by oxygen to maintain various life activities of the body [8]. Normally, the human body utilizes a balanced ROS for the protection of the body. Whenever this balance is tilted, ROS metabolism is disordered and cells are attacked. Cell membranes, organelles, and DNA are damaged, and cardiovascular and cerebrovascular diseases could result from these conditions [9]. When the body is experiencing oxidative stress, it then seeks and takes in external antioxidants to maintain the body's ROS balance, after the internal systems have failed. Our previous work showed that rice residue fermented with *Aspergillus niger* could isolate many small-molecule active peptides and they have good antioxidant activities [10], low sensitivity, and strong biological activity, which can be used as an antioxidant reagent for the body. However, the mechanism of its antioxidant effect is still unclear because in vivo metabolism is complicated. Therefore, it is of great significance to explore the mechanism of active peptide antioxidative action through signaling pathway.

The Keap1-Nrf2/ARE signaling pathway is considered the most important regulatory antioxidant system in the body [11]. Nuclear transcription-related factor 2 (Nrf2) is a key transcription factor that regulates antioxidative stress-related pathway. Under normal physiological conditions, Nrf2 and inhibitor protein Keap1 exist in the cytoplasm in an inactive form. When the cell is in an oxidative stress state, the two become uncoupled, and Nrf2 enters the nucleus, combines with the antioxidant response element (ARE), and activates downstream related gene transcription, thereby protecting the cells from oxidative stress damage [12–14]. Rahman et al. [15] isolated a new type of antioxidant peptide YD1 from *Bacillus*, which acted on RAW264.7 cells and up-regulated heme oxygenase-1 (HO-1) expression through Nrf2 signal pathway. The transcription and translation activities of this factor protect the cell from oxidative damage. The team also found that YD1 reduced the levels of NO and ROS expressions in RAW264.7 cells, resulting in higher levels of antioxidant enzymes in the YD1 treatment group when compared with the oxidative stress group. Tsai et al. [16] isolated active peptide IF from potato and used hypertensive rat model to evaluate antioxidition of the peptide; they found that the Nrf2-dependent antioxidant pathway played a potential protective role in the hypertension-related ROS-mediated renal damage. Based on these experiments, it was speculated that the antioxidant mechanism of active peptide from rice residue mediated through the Nrf2 signaling pathway.

In this study, the peptide F2d (Val-Ala-Glu-Glu-Glu-Leu-Ala-Gly-Asp-Val) with antioxidant activity was isolated and identified from rice residue fermented with *Aspergillus niger* and carried out antioxidant activity analysis *in vitro*. H_2_O_2_-induced HUVEC oxidative injury model was used to evaluate the protective effect of F2d, and the underlying mechanism of F2d exerting its function was investigated. This article provides experimental basis for the role of active peptides from rice residues at the molecular and cellular levels through molecular biology methods, in order to provide new methods for the development and utilization of rice and its by-product and evidences for further research on active peptides from rice residues.

## 2. Materials and Methods

### 2.1. Materials and Reagents

RPMI 1640 and fetal bovine serum were purchased from Gibco-BRL (Gibco-BRL, Carlsbad, California, USA). Active oxygen detection kit (S0033), total SOD active detection kit (S0101), and lipid oxidation detection kit (S0131) were purchased from Full-Style Gold (Beijing, China). The enhanced BCA protein kit (P0009) and nuclear cytoplasmic protein extraction kit (P0028) were purchased from Beyotime Biotechnology (Shanghai, China). Keap1 antibody (catalog number 60027-1-lg), Nrf2 antibody (catalog number 66504-1-lg), NQO1 antibody (catalog number 67240-1-lg), and HO-1 antibody (catalog number 67643-1-lg) are available for purchase from Proteintech (USA). Nrf2 (phospho S40) (catalog number EP1809Y) was purchased from Abcam (UK). *β*-Actin (Cat. No. 12620), anti-rabbit IgG HRP conjugate (V7951), and anti-mouse IgG HRP conjugate (W4021) were purchased from Promega Corporation (Madison, W1, USA). The inhibitor ML385 (catalog number HY-100523) was purchased from MCE (Shanghai, China). All other reagents for this study were of analytical grade.

### 2.2. Preparation and Identification of Rice Residue Active Peptide F2d

*Aspergillus niger* was selected as the best microorganism for fermenting rice residue, and the fermentation conditions such as water content, fermentation time, fermentation temperature, and other conditions of the rice residue were optimized during the fermentation period to obtain the best fermentation process [10]. The fermentation product was separated by ultrafiltration membrane, gel chromatography, and liquid phase separation, and the F2d high antioxidant activity component was collected. The molecular weight determined by MicroTOF-QII tandem mass spectrometry is 1073 Da, and the amino acid sequence is Val-Ala-Glu-Glu-Glu-Leu-Ala-Gly-Asp-Val [17]. Analytical RP-HPLC verified that the active purity is 90.36% [18]. The purity of active peptide which is used in cell experiments usually needs to be more than 98%. The purity and the amount of isolated peptide are not enough to do cell experiments. Synthetic peptide F2ds with the same amino acid sequence and molecular weight were purchased from a biological company.

### 2.3. Determination of Oxidation Resistance of Reduced Iron

According to the report method by Maurya and Chandra with slight modification [19], FRAP reagent was composed of acetate buffer solution (0.3 mol/L, pH 3.6), 10 mmol/L TPTZ solution, and 20 mmol/L ferric chloride solution. The volume ratio of the above solution was 10 : 1 : 1. 3 mL of FRAP reagent was mixed with 20 *μ*L of the sample solution. The mixture was incubated at 37°C for 30 min. FRAP reagent was used to adjust the zero position, and the absorbance of the reactant was measured at a wavelength of 593 nm. The antioxidant activity of the sample was expressed according to the FRAP value. The higher the FRAP value of peptide means the stronger antioxidant activity.

### 2.4. Determination of DPPH Free Radical Scavenging Activity

The method reported by Jiang et al. [20] was used to determine the ability of F2d to remove DPPH^·^ free radicals. 100 *μ*L of the sample solution was added to 2 mL of 0.1 mmol/L DPPH anhydrous methanol solution, the mixture was incubated for 15 min in the dark, and the absorbance was measured at 517 nm. The anhydrous methanol was added to DPPH solution as blank; the anhydrous methanol solution was used to adjust the zero position. The scavenging activity was calculated as follows:

(1) $$\begin{array}{c} \text{DPPH scavenging rate }\left(\%\right)=\frac{\left(A_{\text{C}}-A_{\text{S}}\right)\times 100}{A_{\text{C}}}, \end{array}$$

where *A*_C_ is the absorbance value of the blank and *A*_S_ is the absorbance value of the sample.

### 2.5. Determination of ABTS Free Radical Scavenging Activity

The ABTS method is based on antioxidants to capture the long-lived cationic radical ABTS, which has a blue-green chromophore and characteristic absorption at a wavelength of 734 nm. Antioxidants can transfrom cationic radicals ABTS into ABTS, and the chromospheres change color, and the absorption value is decreased. ABTS cationic radical working solution was prepared as follows: 7 mmol/L ABTS solution and 2.45 mmol/L potassium persulfate solution were mixed and placed at room temperature in the dark for 12-16 h. On the day of the initial measurement, the mixture was diluted with absolute ethanol at 734 nm to 0.70 ± 0.02 AU, which was the ABTS cation radical working solution [21]. The scavenging activity was calculated according to the following formula:

(2) $$\begin{array}{c} \text{ABTS scavenging rate }\left(\%\right)=\frac{\;\left(A_{\text{C}}-A_{\text{S}}\right)\times 100}{A_{\text{C}}}, \end{array}$$

where *A*_C_ is the absorbance value of the blank and *A*_S_ is the absorbance value of the sample.

### 2.6. Measurement of Intracellular ROS Levels

A reactive oxygen species analysis kit was used to measure H_2_O_2_-induced oxidative stress. First, the cells were cultured to 4 × 10^4^/mL according to the method requirements of the kit, and F2d and H_2_O_2_ were added in groups according to the experiment. The 2,7-dichlorodi-hydrofluorescein diacetate (DCFH-DA) solution was diluted with serum-free medium to a final concentration of 10 *μ*mol/L. Then, the original culture medium was aspirated, DCFH-DA solution was added to cover the cells, and the cells were incubated in a CO_2_ incubator at 37°C for 30 min. Then, the DCFH-DA solution was aspirated, the cells were washed 3 times with serum-free medium, and the fluorescence intensity was detected with a microplate reader under the conditions of excitation wavelength of 488 nm and emission wavelength of 525 nm. In order to make the experiment more accurate, we consider adding detection of other oxidation indicators. Therefore, the superoxide dismutase (SOD) and malondialdehyde (MDA) were detected by related kits (Full-Style Gold, Beijing, China).

### 2.7. Cell Culture

HUVEC was purchased from the Institute of Cell Resource Center, Chinese Academy of Sciences (Shanghai); the cell line originated from the ATCC (American Type Culture Collection, United States). HUVEC was incubated with 10% fetal bovine serum (FBS), 100 U/mL streptomycin, and 100 U/mL penicillin at 37°C and 5% CO_2_. F2d was dissolved in the culture medium. HUVEC cells were cultured in a 10 cm Petri dish and treated as follows: control group, H_2_O_2_ treatment group, H_2_O_2_+F2d (12.5 *μ*g/mL, 25 *μ*g/mL, and 50 *μ*g/mL) group, or H_2_O_2_+F2d (50 *μ*g/mL)+Nrf2 inhibitor (ML385) group. After the cell culture was completed, cells from different treatment groups were collected separately.

### 2.8. Determination of Cell Viability

0.25% trypsin was used to digest the well-growing HUVEC cells and prepare cell suspension, a complete medium containing 10% FBS and 1640 medium was added to cell suspension, and then, the cells were inoculated in 96-well plates. Waiting for the cells to grow to 80%, F2d was added to make the final concentrations of F2d which were 12.5 *μ*g/mL, 25 *μ*g/mL, and 50 *μ*g/mL, respectively. And then, a blank control group and H_2_O_2_ treatment groups (20, 40, 60, 80, and 100 *μ*mol/L) were established. After that, 10 *μ*L of MTS solution was added to each well and placed in a CO_2_ incubator for 30 min. The absorbance was measured at 492 nm within 15 min by using an enzyme-linked immunoassay analyzer. The cell survival rate was expressed as a percentage of the control value, and the experiment was repeated three times and averaged.

### 2.9. Cell Morphology and Apoptosis Analysis

In order to observe the treatment of H_2_O_2_-induced HUVEC injuries using F2d, we seeded the cells in 96-well plates and established experimental groups after the cells adhered and added corresponding concentrations of F2d and H_2_O_2_. After 24 h of culture, the cell morphology was observed using an inverted microscope and then photographed.

The level of apoptosis was detected by flow cytometry using FITC Annexin V Apoptosis Detection Kit (BD, USA). HUVECs were inoculated into Petri dishes at 4 × 10^4^/well and cultured overnight. Cells were collected, resuspended in PBS (4°C), and centrifuged at 2000 rpm for 10 min; 300 *μ*L of 1× binding buffer and 5 *μ*L of Annexin V-FITC were added to the precipitate. The above solution was mixed and placed at room temperature in the dark for 15 min, 5 *μ*L of PI staining and 200 *μ*L of 1× binding buffer were added after 5 min, and the BD Accuri C6 flow cytometer (BD, USA) was used for apoptosis analysis.

### 2.10. Total RNA Extraction and Quantitative RT-PCR

According to the method of Guo et al. [22], real-time quantitative fluorescence PCR instrument (C1000 Touch™ Thermal Cycler) was used to detect the expression level of key target genes such as Nrf2. HUVEC cells in good condition in the logarithmic growth phase were inoculated in six-well plates after digestion; active peptide F2d was added to make the final concentration of F2d 0 *μ*g/mL, 12.5 *μ*g/mL, 25 *μ*g/mL, and 50 *μ*g/mL; and H_2_O_2_ was added; the total RNA was extracted according to the instruction manual of TRIzol kit after 12 h. The RNA concentration was measured using NanoDrop, RNA integrity test was conducted to ensure the quality of the extracted RNA, and reverse transcription kit was used to synthesize cDNA. A conventional PCR reaction system was used to conduct experiments using a RT-PCR instrument. According to the 2^-△△Ct^ (RQ) method, the relative expression of the target gene mRNA was calculated. Primers were designed using Primer 5.0 software and synthesized by the company: *β*-actin upstream primer: 5′-CTC CTC CCT GGA GAA GAG CTA C-3′, *β*-actin downstream primer: 5′-TGA TGG AGT TGA AGG TAG TTT CG-3′; Bax upstream primer: 5′-TTT GCT TCA GGG TTT CAT CCA-3′, Bax downstream primer: 5′-GAG ACA CTC GCT CAG CTT CTT G-3′; Bcl-2 upstream primer: 5′-GTG CCT GCT TTT AGG AGA CCG A-3′, Bcl-2 downstream primer: 5′-GAG ACC ACA CTG CCC TGT TGA-3′.

### 2.11. Western Blot

The experiment was conducted according to the method used in the previous study [23]. HUVEC cells in the logarithmic growth phase were inoculated into a 10 cm Petri dish, added with active peptide F2d, and divided into 0 *μ*g/mL, H_2_O_2_ group, 12.5 *μ*g/mL, 25 *μ*g/mL, and 50 *μ*g/mL according to the concentration. Groups were cocultured for 16 h, and then, the total protein of the cells was extracted using RIPA buffer solution, and the protein concentration was determined using BCA kit. The cell protein was separated by 10%-15% SDS-PAGE gel electrophoresis, and the protein was transferred to the PVDF membrane in 60 V voltage transfer solution and then put membrane in blocking solution (5% skim milk powder) for 1 h-2 h. The membrane incubated with *β*-Actin (1 : 5000; Promega Corporation, USA), Nrf2, Keap1, HO-1, NQO1, Bax, Bcl-2 (1 : 2500; Proteintech, USA), and Nrf2 (phospho S40) (1 : 5000; Abcam, United Kingdom). After overnight at 4°C, the membrane was washed with TBST three times at room temperature for 10 min each time and then incubated with 1 : 10000 mouse/rabbit secondary antibody for 1 h. Wash with TBST three times for 10 min each time. The immune response was detected by ECL Plus™ Western Blotting Detection System (Pierce, Rockford, USA), and the protein was imaged in a gel imaging system (ChemiDoc™ XRS^+^, Bio-Rad). Finally, ImageJ software was used to calculate the gray value of each protein band to determine the relative expression of target protein and control protein.

### 2.12. Statistical Analysis

All experiments were carried out in triplicate and normalized the data to the level of the control in each sample. The experimental data were analyzed by SPSS 13.0 software (SPSS, Chicago, Illinois, USA). All data were expressed as mean ± standard deviation (SD) and analyzed using one-way ANOVA and the Tukey test for comparison between groups. The chi-square test is used for percentage (ratio) comparison. A statistical difference at *P* value less than 0.05 was considered significant.

## 3. Results

### 3.1. F2d Amino Acid Sequence and Purity Identification

**Figure 1:**
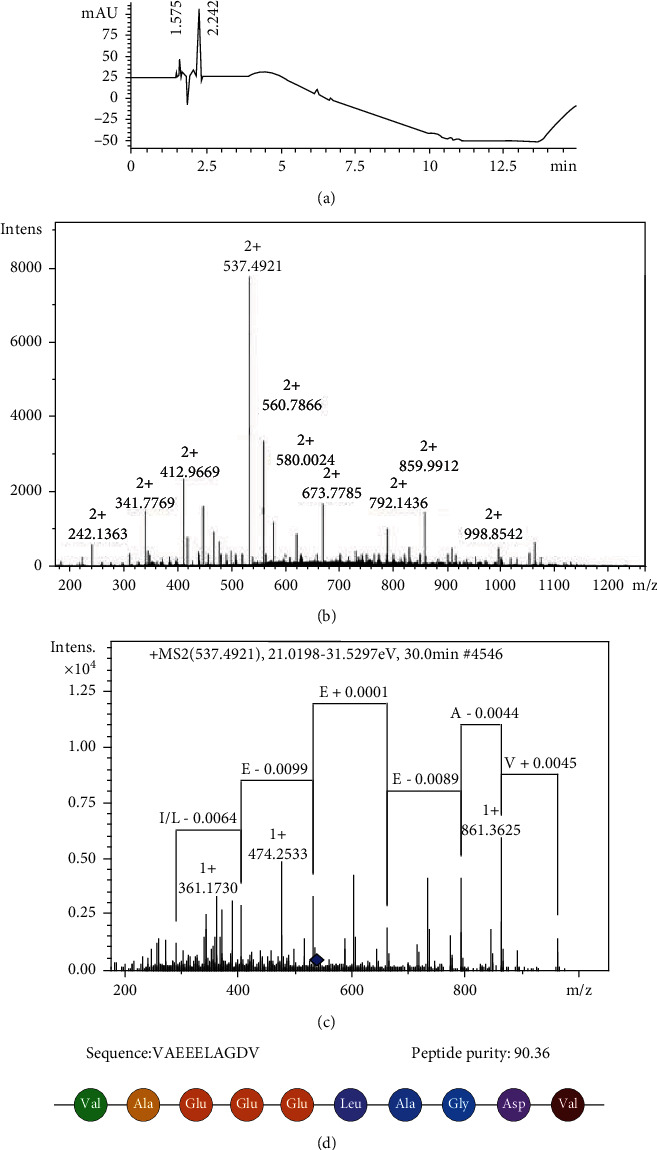
F2d amino acid sequence and purity identification. (a) Peptide purity identified using RP-HPLC analysis. Run time: 15 min; wavelength: 210 nm; flow rate: 1 mL/min. (b) MS1 analyzes the molecular weight of F2d. (c) MS2 analyzed the structural identification of F2d. (d) Sequence of peptide F2d.

RP-HPLC was used to analyze the purity of the F2d component. As shown in Figure 1(a), there were a few impurity peaks in F2d; the peak area of F2d reached 90.36%, which had little effect on subsequent structure identification. The molecular weight of the antioxidant component F2d was determined by MicroTOF-QII tandem mass spectrometer MS1 (Figure 1(b)). The molecular weight of F2d was 1072.9686 Da. The molecular structure of F2d was determined by secondary mass spectrometry (Figure 1(c)) and analyzed by De Novo software. F2d is a 10-amino acid peptide with the sequence Val-Ala-Glu-Glu-Glu-Leu-Ala-Gly-Asp-Val.

### 3.2. F2d Antioxidant Capacity

**Figure 2:**
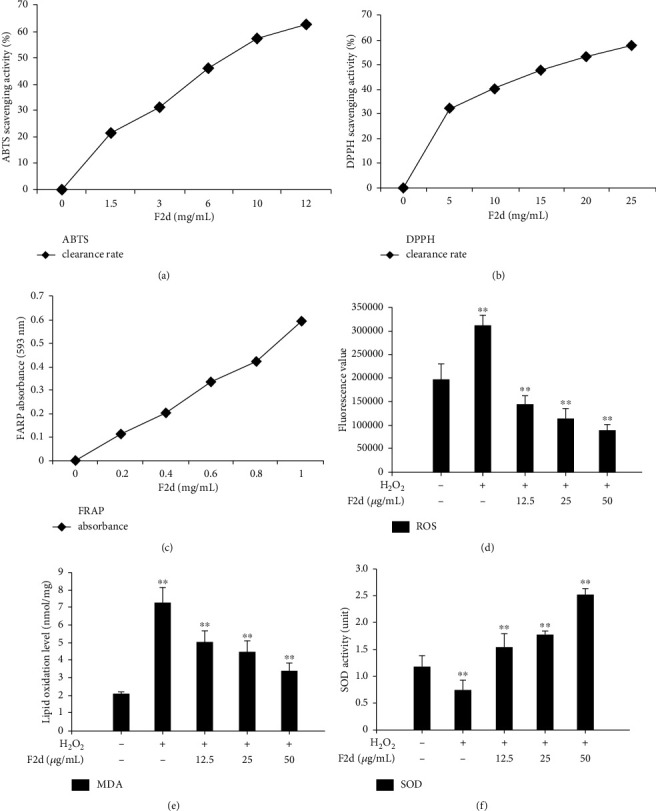
F2d antioxidant capacity test. The scavenging activity of F2d on ABTS and DPPH free radicals was measured (a, b), and the antioxidant activity of F2d was detected by FRAP (c). The effect of different concentrations of F2d on the changes of ROS (d), MDA (e), and SOD (f) in HUVECs induced by H_2_O_2_. The data are presented as the mean ± SD of three independent experiments. ^∗∗^*P* < 0.01.

We have studied the antioxidant activity of F2d; the experimental methods are DPPH method, ABTS method, and FRAP method. The results showed that F2d exerted its ability to scavenge free radicals in a dose-dependent manner (Figures 2(a) and 2(b)), and FRAP results also proved that F2d enhanced its antioxidant capacity as the concentration gradient increased (Figure 2(c)). Zheng et al. [24] found that collagen peptides showed strong scavenging activity against oxygen radicals in their study. In this study, we also measured the change trends of the main oxidation indicators ROS, MDA, and SOD after H_2_O_2_ and F2d treatments (Figures 2(d)–2(f)). The results showed that F2d can reduce the increase in ROS levels caused by H_2_O_2_, reduce the upward trend of MDA, and increase the level of SOD. These data can prove that F2d can reduce the damage of H_2_O_2_ to cells and has strong antioxidant capacity. Because only a small amount of F2d peptide was isolated and purified from fermenting rice residue, synthesized F2d peptide was used for further experiments. The results showed that there was no significant difference in antioxidant capacity between the isolated and synthesized F2d peptides through DPPH and ABTS assays (see Suppl Figure 1).

### 3.3. The Effect of F2d on the Oxidative Damage of HUVECs

When H_2_O_2_ induces cell damage, HUVEC cell morphology will obviously deteriorate. In this study, the control group cells were spindle-shaped, cells grew well and adhered firmly, and cells were tightly connected. In the H_2_O_2_ treatment group, the cell morphology became shrink and round, and the intercellular spaces were enlarged, but the cell boundaries were clear, and the morphology tended to apoptosis, showing obvious injury morphology. In the H_2_O_2_ and F2d cotreatment group, with the increase of F2d concentration, the cell morphology gradually returned to normal. The cell morphology of the 50 *μ*g/mL treatment group was similar to that of the control group. Therefore, it can be seen from the cell morphology results that F2d has an inhibitory effect on HUVEC apoptosis induced by H_2_O_2_.

**Figure 3:**
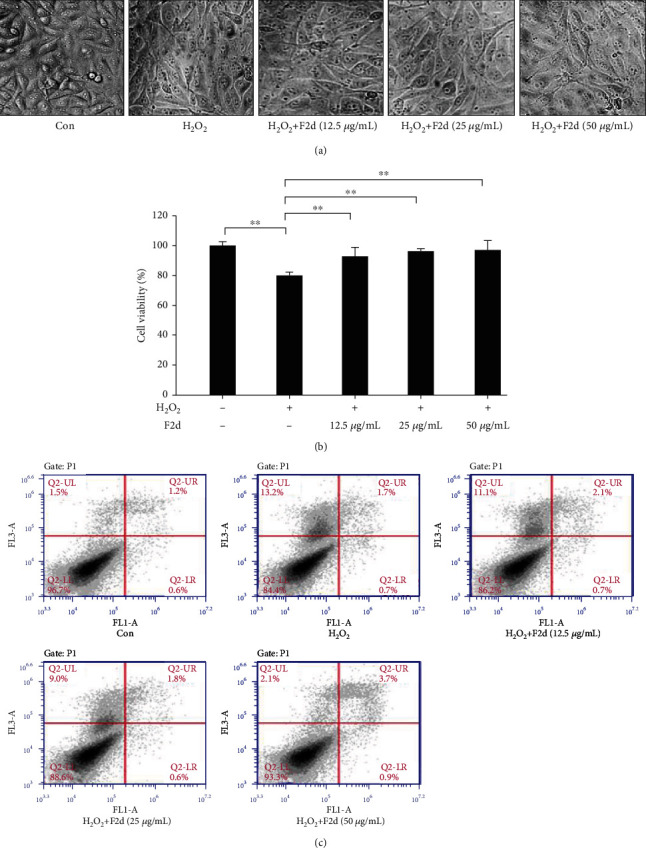
The effect of F2d on the oxidative damage of HUVECs. The effects of different concentrations of F2d on HUVEC morphology after H_2_O_2_ induction were observed with a microscope (a); the effects of different concentrations of F2d on the cell viability of HUVECs were determined by MTS assay (b); the apoptosis of HUVECs in the five indicated groups was determined by flow cytometry (c). The data are presented as the mean ± SD of three independent experiments. ^∗∗^*P* < 0.01.

In this study, the concentration of 60 *μ*M H_2_O_2_ was used to establish oxidative damage model in HUVEC cells, and it was processed as follows: control group, H_2_O_2_ treatment group, and H_2_O_2_+F2d (12.5 *μ*g/mL, 25 *μ*g/mL, and 50 *μ*g/mL); cell viability was detected by MTS method to evaluate the effect of F2d on H_2_O_2_ oxidative damage. The MTS results showed (Figure 3(b)) that the cell viability of the H_2_O_2_ group decreased, and the F2d concentration was increased, and the cell viability increased. The isolated peptides were also evaluated for their protective effect on H_2_O_2_ oxidative damage by MTS assay in HUVECs, and no significant difference was found between the isolated and synthesized F2d peptides. Hippocampal-derived peptides have been shown to have good cytoprotective activity [25]. F2d exhibited better cytoprotective capacity. Therefore, F2d has a protective effect on oxidative damage caused by H_2_O_2_.

In order to further prove the effect of F2d on HUVEC cell apoptosis, flow cytometry was used to detect the distribution ratio of cells in each period. As shown in Figure 3(c), H_2_O_2_ treatment can significantly increase the percentage of HUVEC cell apoptosis, and F2d treatment can reduce the ratio of H_2_O_2_-induced apoptosis and necrotic cells. From this, we concluded that F2d can reduce HUVEC cell apoptosis induced by H_2_O_2_.

### 3.4. F2d Inhibits Oxidative Damage via the Nrf2 Signaling Pathway

**Figure 4:**
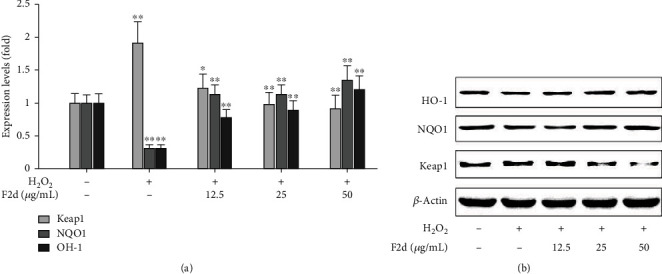
Expression of HO-1, NQO1, and Keap1 proteins in F2d against oxidative injury in HUVECs. Protein levels of antioxidant proteins HO-1, NQO1, and Keap1 in the H_2_O_2_ and F2d+H_2_O_2_ groups of the control group were determined by western blot (a, b). The data are presented as the mean ± SD of three independent experiments. ^∗^*P* < 0.05; ^∗∗^*P* < 0.01.

**Figure 5:**
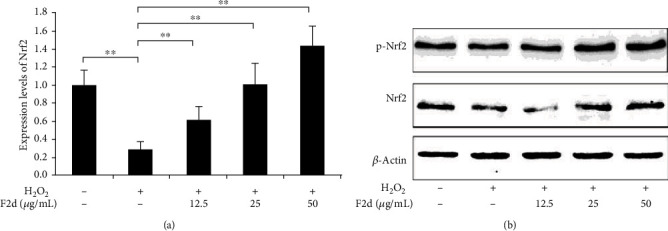
F2d increased Nrf2 activation in H_2_O_2_-induced HUVECs. The expression level of antioxidant Nrf2 and its kinase activation was determined by western blot (a, b). The data are presented as the mean ± SD of three independent experiments. ^∗∗^*P* < 0.01.

**Figure 6:**
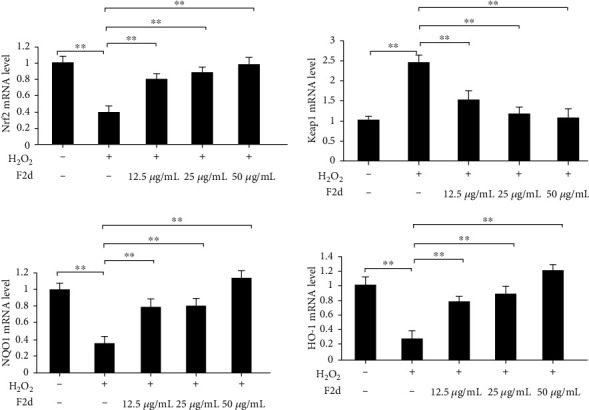
F2d regulates antioxidant-related genes and attenuates oxidative damage in HUVEC. F2d detects the expression of antioxidant-related genes by quantitative RT-PCR. The data are presented as the mean ± SD of three independent experiments. ^∗∗^*P* < 0.01.

Nrf2 is the most important endogenous antioxidant signaling pathway in the body. Therefore, we speculated whether the mechanism of F2d showing strong antioxidant activity is related to Nrf2 regulation of oxidative damage. We detected the expression of Nrf2 and its related genes in HUVEC after H_2_O_2_ and F2d treatment. Western blot results showed that (Figures 4(a) and 4(b)) H_2_O_2_ could downregulate the protein expression of downstream Nrf2 and increase the protein expression of Keap1. F2d can greatly reduce the effect of H_2_O_2_ on protein expression, and the effect becomes more obvious with the increase of concentration. The expression of Nrf2 in vivo is not only inhibited by Keap1, but also phosphorylation of Nrf2 is a way of activation. The important role of Nrf2 phosphorylation in the antioxidant mechanism has been identified in Liu et al.'s studies on Ginkgo biloba extracts [26]. A similar mechanism of action of F2d was also demonstrated in the present study. As shown in Figures 5(a) and 5(b), under the action of F2d, the mRNA and protein expression of Nrf2 increased, while the protein expression level of p-Nrf2 increased, and the kinase activity also increased. With the increase of F2d concentration, the phosphorylation of Nrf2 showed an increasing trend. Based on these data, we can confirm that F2d can increase the expression of antioxidant genes of Nrf2 and its downstream NQO1 and HO-1. It may act by inhibiting the expression of Keap1 while upregulating the phosphorylation of Nrf2. To further verify the molecular mechanism of F2d attenuating H_2_O_2_-induced oxidative damage in HUVEC, the mRNA expression levels of Nrf2, Keap1, NQO1, and HO-1, key genes of the Nrf2 signaling pathway, were examined. RT-PCR results showed that F2d could promote the expression of antioxidant genes Nrf2, NQO1, and HO-1 and decrease the Keap1 mRNA expression (Figure 6). This proved that the molecular mechanism of F2d's antioxidant action may be accomplished through the Nrf2 signaling pathway. More experiments are needed to verify the existence of other signaling pathways.

### 3.5. F2d Regulates Apoptosis-Related Genes and Inhibits HUVEC Apoptosis

**Figure 7:**
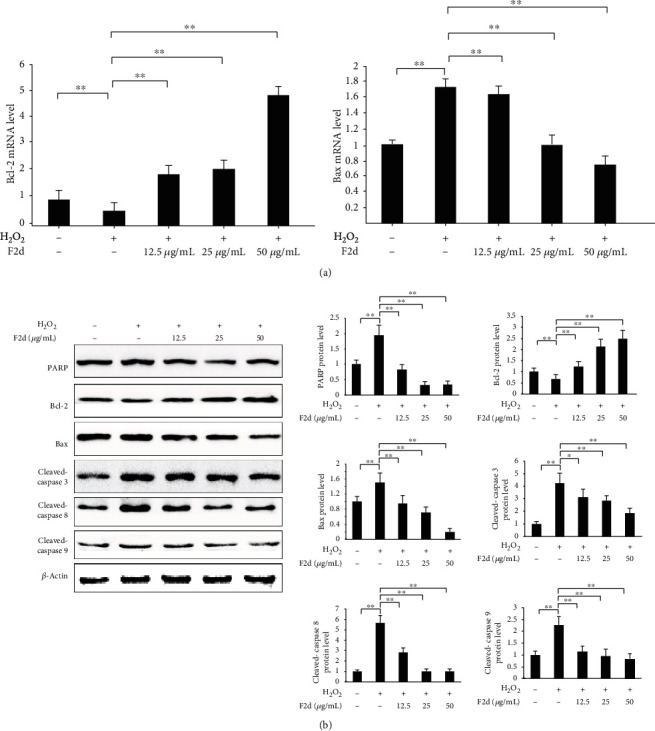
F2d regulates apoptosis-related genes and inhibits HUVEC apoptosis. F2d detected the expression of Bax and Bcl-2 apoptosis-related genes by quantitative RT-PCR (a). F2d detected the expression of apoptosis-related proteins by western blot (b). The data are presented as the mean ± SD of three independent experiments. ^∗∗^*P* < 0.01; ^∗^*P* < 0.05.

In order to further explore the molecular mechanism of F2d attenuating H_2_O_2_ inducing HUVEC apoptosis, mRNA and protein expression levels of apoptosis-related proteins Bcl-2, Bax, Caspase-3, Caspase-8, and Caspase-9 were detected. Previous studies have demonstrated that active peptides of the same source regulate apoptotic genes to suppress the level of apoptosis after oxidative damage to cells [27]. RT-PCR results showed that F2d up-regulated the mRNA expression of Bcl-2 and reduced the mRNA expression of Bax (Figure 7(a)). Western blot results showed that Bcl-2 expression increased and Bax expression decreased (Figure 7(b)). Compared with the control group, the ratio of Bax/Bcl-2 was significantly upregulated after H_2_O_2_ treatment, but the ratio could be reduced by F2d pretreatment, which proved that the result was reliable. On the other hand, the protein expression of Caspase-3, Caspase-8, and Caspase-9 in the H_2_O_2_ damaged group was significantly increased, and F2d treatment inhibited the influence of H_2_O_2_ on the protein level. The experimental data can be summarized to prove that F2d has a protective effect on H_2_O_2_-induced HUVEC by inhibiting mitochondrial-mediated signaling pathway and Caspase-3 apoptotic signaling pathway.

### 3.6. Blocking the Nrf2 Pathway Inhibits the Protective Function of F2d

**Figure 8:**
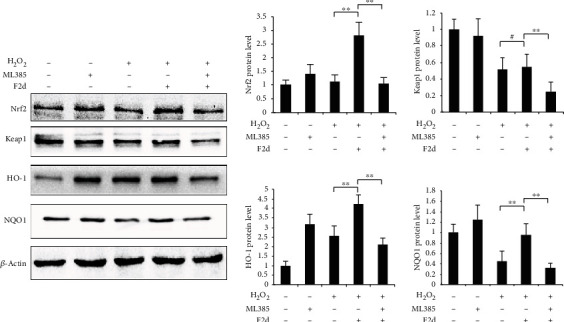
Blocking the Nrf2 pathway inhibits the protective function of F2d. Western blotting assay was used to determine the control, ML385, H_2_O_2_, F2d+H_2_O_2_, and F2d+H_2_O_2_+ML385 group of antioxidant signal pathway-related protein expression. The data are presented as the mean ± SD of three independent experiments. ^∗∗^*P* < 0.01; ^#^*P* > 0.05.

**Figure 9:**
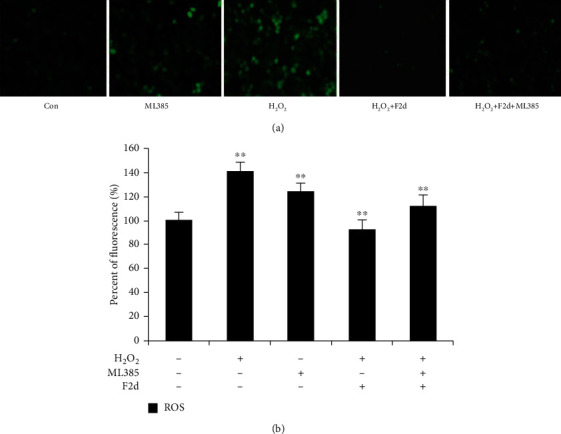
Blocking the Nrf2 signal pathway to detect ROS content. Intracellular ROS in HUVECs indicated as green fluorescence by DCFH-DA (a). Under conditions of H_2_O_2_-induced oxidative stress of HUVEC cells, ROS content was detected after ML385 blocked Nrf2 (b). The data are presented as the mean ± SD of three independent experiments. ^∗∗^*P* < 0.01.

In order to prove that F2d exerts its antioxidant effect through the Nrf2 signaling pathway, we decided to add the Nrf2 specific inhibitor ML385 to block its expression and detect the changes in the expression levels of Nrf2-related genes and the oxidative stress index ROS after blocking. It has been shown that ML385 blocks the protective mechanism of Nrf2 *in vivo*, proving that the natural ingredient has good physiological activity [28]. The experimental results showed (Figure 8) that when F2d was treated with H_2_O_2_, the expression levels of Nrf2 and its downstream genes were significantly reduced after the inhibitor ML385 was added, which proved that ML385 blocks the protein expression of Nrf2 and downstream genes were also affected. Most importantly, the ROS level detection results were in line with expectations. As shown in Figure 9(a), ROS levels in HUVEC treated with H_2_O_2_ increased significantly, while F2d- (50 *μ*g/mL) treated cells for 12 h could significantly reduce H_2_O_2_-induced ROS production, and ROS levels increased slightly after the addition of inhibitor ML385. In order to show the level of ROS in cells more intuitively, the cells were observed with a fluorescence microscope (Figure 9(b)). The control group showed weak green fluorescence, while the green fluorescence intensity of the ML385 and H_2_O_2_ treatment groups was significantly increased. The H_2_O_2_ treatment group had the highest green fluorescence intensity. After F2d pretreatment, the fluorescence intensity was significantly reduced, while the addition of ML385 showed weak fluorescence. From this, we concluded that F2d has a strong antioxidant effect. After ML385 blocks Nrf2, the antioxidant effect of F2d is significantly weakened, but it is not completely invalid. Finally, we can confirm the basic theory that F2d exerts its antioxidant effect through Nrf2, but it may also reflect antioxidant activity through other signal pathways, which requires further research in subsequent experiments.

## 4. Discussion

Rice residue is a by-product of rice processing, and its rich protein content makes it a raw material for the extraction of antioxidant components. Dr. Tian Wei, a researcher of our group, has prepared antioxidant products by selecting rice fermented by *Aspergillus niger*. Therefore, we synthesized the small-molecule active peptide F2d (Val-Ala-Glu-Glu-Glu-Leu-Ala-Gly-Asp-Val) through the amino acid sequence provided by the research team and verified the purity by the RP-HLPC method and the method of using MicroTOF-QII tandem mass spectrometer to ensure the accuracy of amino acid sequence. At the same time, we conducted F2d antioxidant capacity tests (ABTS, DPPH, and FRAP) [29] and proved that as the concentration of active peptide increased, the ability to scavenge free radicals showed an upward trend. Furthermore, we also studied the detection of oxidation index (ROS, MDA) and antioxidant index (SOD) [30] and obtained the same results as the previous experiment. Finally, we have concluded that the active peptide F2d has good antioxidant activity.

In order to further study the protective function of F2d on oxidative stress injury, we selected HUVEC induced by H_2_O_2_ as the cell model to study the effect of F2d on apoptosis. It should be noted that before exploring the protective effect of F2d, we need to do MTS experiments to measure cell activity as an indicator of cell damage. The MTS test is a commonly used method for detecting cytotoxicity [31]. The results show that three concentrations of F2d have no toxic effect on cell activity, which is the experimental premise of our research. In endothelial cells, H_2_O_2_ stimulates cells to produce excess ROS, which puts cells in a state of oxidative stress and is a classic cell model to evaluate antioxidant ability of active peptides [27]. Therefore, we used H_2_O_2_ to induce HUVEC to produce oxidative stress as a cell model to study the effect of F2d on H_2_O_2_-induced apoptosis and its underlying mechanism. In this study, HUVEC after H_2_O_2_ induction showed a significant damage state compared to normal cells, and with the increase of H_2_O_2_ concentration, cell viability gradually decreased, and the final concentration of H_2_O_2_, which was the earliest cell damage, was determined to be the modeled concentration of 60 *μ*mol/L. Subsequent experiments proved that, at this modeled concentration, F2d at a dose of 12.5 *μ*g/mL can significantly reduce the cell damage induced by H_2_O_2._ When the concentration of F2d was 50 mg/mL, the cell survival rate reached the maximum value. Therefore, we conclude that F2d has a significant protective effect on H_2_O_2_-induced HUVEC oxidative damage. On this basis, we continue to further study the molecular mechanism of F2d inhibiting apoptosis.

In endothelial cells, hydrogen peroxide can stimulate signaling pathway activation and produce ROS. A large number of studies have shown that ROS plays a very important role in oxidant-induced apoptosis [32–34]. The accumulation of ROS can lead to lipid peroxidation, impaired mitochondrial membrane structure and function, and the dissipation of proton electrochemical gradients, which ultimately leads to apoptosis [35]. In order to further study the functional role of F2d on oxidative stress injury, we selected HUVEC induced by H_2_O_2_ as the cell model and determined the expression levels of apoptosis-related proteins Bcl-2, Bax, and Caspase-3 to study the effect of F2d on apoptosis influences. The latest research reports showed that Keap1, a cytoplasmic inhibitory protein that regulated Nrf2, also played a very important role in antiapoptosis [36]. On the basis of this theory, first, we detected the apoptosis after F2d treatment by flow cytometry. It was found that the H_2_O_2_ group showed the most severe degree of apoptosis, but the degree of apoptosis was gradually reduced after F2d treatment, so we concluded that F2d can inhibit apoptosis and reduce oxidative damage of cells. At the same time, we consulted the literature and found that the Nrf2 pathway has a direct role in regulating apoptosis [37]. Studies have found that Keapl is related to apoptosis-related proteins PGAM5, FAC1, p26, and SQSTM1, which suggests that F2d may play its antioxidative role in between the Nrf2 pathway and the apoptosis pathway. There is an important connection, in which the combination of PGAM5 and Keapl is a direct regulator of the Nrf2 pathway and the apoptosis pathway [38]. Niture and Jaiswal [ 39] pointed out that Keapl can combine with Bcl-2, the expression of Bax increased, and caspase was activated to induce apoptosis. In our study, we also found that treatment of HUVEC with different concentrations of F2d significantly inhibited the protein expression of Keap1, Bax, and Caspase3 and also promoted the expression of Nrf2 and Bcl-2, ensuring cell stability and cell survival. The above research results showed that F2d plays a protective role against H_2_O_2_ damage in HUVEC through the inhibition of apoptosis.

Based on the above research and analysis, we further studied the antioxidant mechanism of F2d. Nrf2 is a key factor in cellular oxidative stress response [40]. Under normal physiological conditions, Nrf2 is located in the cytoplasm by the actin-binding inhibitor protein Keap1 (a zinc metal protein located near the plasma membrane); under oxidative stress, Nrf2 is isolated from the cytoplasm Keap1 and is alkaline; released from Keap1 in the cytoplasm, and transferred to the nucleus, which targets the antioxidant response element (ARE) to activate the transcription of downstream genes and then translates a series of related proteins to play physiological functions [41–43]. Since the Keap1-Nrf2 pathway plays a central role in oxidative stress, a deep understanding of the mechanism by which Keap1 senses and responds to H_2_O_2_ is needed. In our study, under the condition that hydrogen peroxide induces HUVEC to establish an antioxidant model, western blot experimental results showed that Keap1 expression was upregulated, and the addition of F2d made Nrf2 show a trend of upregulation with concentration gradient, and Keap1 had the opposite trend. Therefore, we boldly speculated that there was a mutual regulation relationship between Nrf2 and Keap1. Some literature has pointed out that the increase of Nrf2 level will promote the binding with antioxidant response element (ARE), so that free Nrf2 translocates into the nucleus and activates the transcription of downstream genes [44]. The experimental results in this study also confirmed the view that F2d pretreatment increased the expression of Nrf2 protein, entered the nucleus, and activated a series of ARE-dependent gene expression of antioxidant and cytoprotective proteins, such as HO-1 and NQO1. Nonetheless, Nrf2 may be regulated by other factors, and these factors may also affect Keap1 levels, and Nrf2 has other regulatory mechanisms. Protein phosphorylation is a key step in regulating cell signaling and the main mechanism of protein posttranslational modification. Phosphorylation of Nrf2 is another major mode of Nrf2 activation. There are several protein kinases such as mitogen-activated protein kinase (MAPK), protein kinase C (PKC), pancreatic endoplasmic reticulum kinase (PERK), and phosphatidylinositol-3-kinase (PI3K) that can participate in its activation, resulting in a weakened antioxidant response mediated by Nrf2 [45]. In this experiment, we proved that with the increase of F2d concentration, the degree of Nrf2 phosphorylation showed an upward trend, indicating that Nrf2 may regulate the entire signaling pathway through phosphorylation, and also proved that F2d plays an antioxidant role through phosphorylation activation. ML385 interacts with Nrf2, affects the DNA binding activity of the Nrf2-MAFG protein complex, and is a novel and specific Nrf2 inhibitor [46]. In order to further determine that F2d exerts its antioxidant effect through the Nrf2 signaling pathway, we added an Nrf2 inhibitor experiment on the basis of the original experiment. Experiments have shown that when Nrf2 expression is inhibited, downstream antioxidant genes are also suppressed, while Keap1 expression is increasing. Combined with the comparison of ROS content before and after the addition of ML385, when Nrf2 was inhibited by ML385, the ROS content increased significantly, which had no protective effect on oxidative damage to cells. From this, we conclude that the antioxidant effect of F2d is accomplished through the Nrf2 signaling pathway. But whether it still works through other channels has yet to be verified.

Summarizing our research results, we confirmed the antioxidant activity of the active peptide F2d extracted by fermented rice residue, and it has a protective effect on the oxidative damage caused by H_2_O_2_-induced HUVEC cells, which may be inevitably related to the regulation of the Nrf2 signaling pathway. These findings further promote the development of by-product biological functions after deep processing of rice and deepen the understanding of the theoretical basis required for the mechanism of action of small-molecule active peptides. These studies are necessary to further study the molecular mechanism of antioxidation for the production of functional products of rice residue active peptides.

## Data Availability

The data used to support the findings of this study are included within the article.

## Abbreviations

ARE: Antioxidant responsive elements
F2d: Rice residue small-molecule active peptide
H_2_O_2_: Hydrogen peroxide
HO-1: Heme oxygenase-1
HUVEC: Human umbilical vein endothelial cell
Keap1: Kelch-like ECH-associated protein 1
MDA: Malondialdehyde
ML385: Nrf2 specific inhibitor
NQO1: NADH quinone oxidoreductase 1
Nrf2: Nuclear factor erythroid-2-related factor 2
ROS: Reactive oxygen species
SOD: Superoxide dismutase.

## Supplementary Materials

Supplementary 1 Suppl Figure 1: comparing the antioxidant capacities of 2 types (isolated and synthetic) of F2d peptides. (A) The scavenging activities of F2d (isolated and synthetic) on ABTS; 7 mmol/L ABTS solution and 2.45 mmol/L potassium persulfate solution were mixed and placed at room temperature in the dark and added different concentrations of isolated and synthetic F2d peptides. The absorbance was measured at 734 nm. The scavenging activities of isolated and synthetic F2d peptides were calculated according to Materials and Methods, Section 2.5 protocol. (B) The scavenging activities of F2d (isolated and synthetic) on DPPH. Different concentrations of isolated and synthetic F2d peptide (100 *μ*L) solution were added to 2 mL of 0.1 mmol/L DPPH anhydrous methanol solution, the mixture was incubated for 15 min in the dark, and the absorbance was measured at 517 nm. The scavenging activities isolated and synthetic F2d peptides were calculated according to Materials and Methods, Section 2.4 protocol.

Supplementary 2 Suppl Figure 2: the effects of different types of F2d peptides (isolated and synthetic) on the cell viability of HUVECs were determined by MTS assay. The isolated or synthetic F2ds were added to make the final concentrations of F2d which were 12.5 *μ*g/mL, 25 *μ*g/mL, and 50 *μ*g/mL, respectively. After 2 h, HUVEC cells were added H_2_O_2_ treatments. 10 *μ*L of MTS solution was added to each well and placed in a CO_2_ incubator for 30 min. The absorbance was measured at 492 nm by using an enzyme-linked immunoassay analyzer. According to the OD value, cell viabilities of different peptides were assessed.
