# Supplementary figures and images for: Bioactive Peptide F2d Isolated from Rice Residue Exerts Antioxidant Effects via Nrf2 Signaling Pathway
Source: Oxid Med Cell Longev. 2021 Sep 29;2021:2637577. doi: 10.1155/2021/2637577 (PMC8495468; doi:10.1155/2021/2637577)

**A**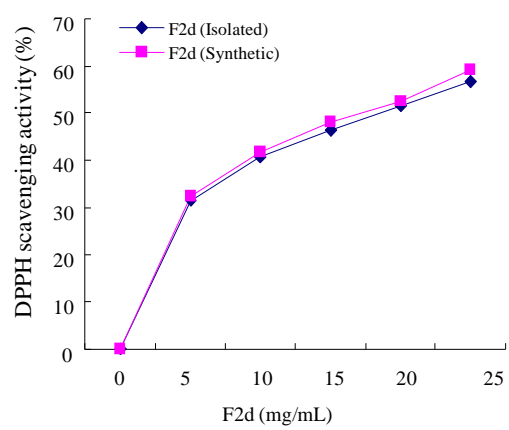**B**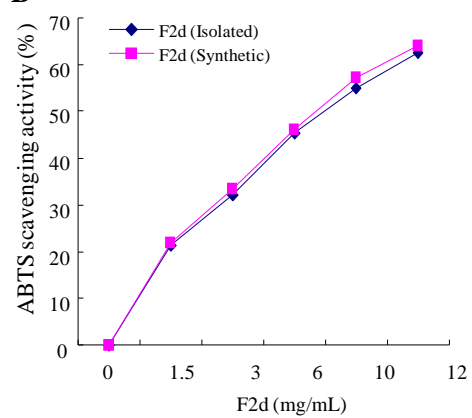

Supplement: Supplementary 1 — Suppl Figure 1: comparing the antioxidant capacities of 2 types (isolated and synthetic) of F2d peptides. (A) The scavenging activities of F2d (isolated and synthetic) on ABTS; 7 mmol/L ABTS solution and 2.45 mmol/L potassium persulfate solution were mixed and placed at room temperature in the dark and added different concentrations of isolated and synthetic F2d peptides. The absorbance was measured at 734 nm. The scavenging activities of isolated and synthetic F2d peptides were calculated according to Materials and Methods, Section 2.5 protocol. (B) The scavenging activities of F2d (isolated and synthetic) on DPPH. Different concentrations of isolated and synthetic F2d peptide (100 μL) solution were added to 2 mL of 0.1 mmol/L DPPH anhydrous methanol solution, the mixture was incubated for 15 min in the dark, and the absorbance was measured at 517 nm. The scavenging activities isolated and synthetic F2d peptides were calculated according to Materials and Methods, Section 2.4 protocol. [file 2637577.f1.pdf]

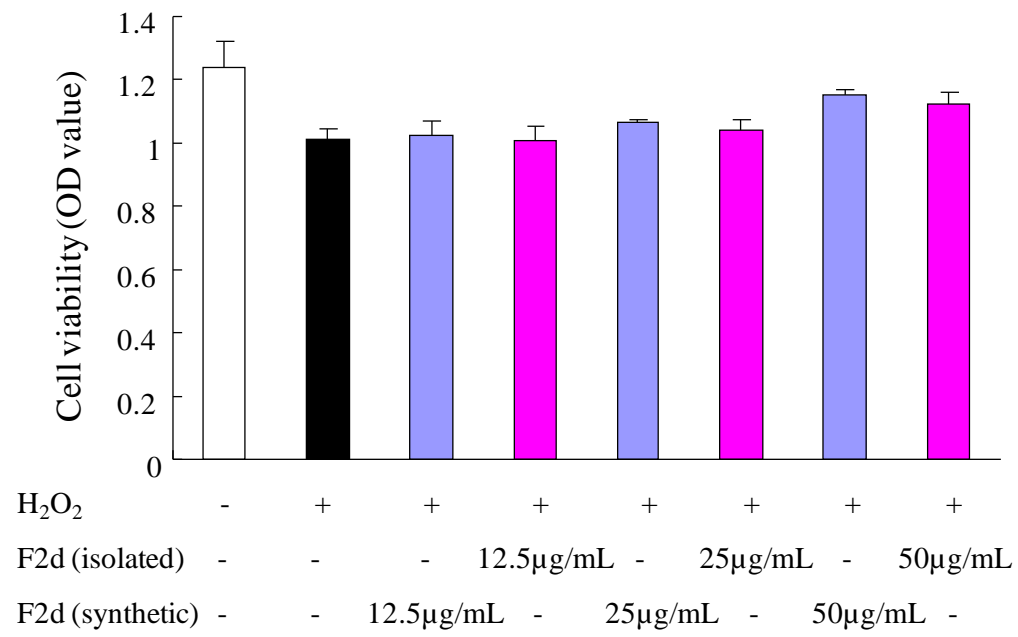

Supplement: Supplementary 2 — Suppl Figure 2: the effects of different types of F2d peptides (isolated and synthetic) on the cell viability of HUVECs were determined by MTS assay. The isolated or synthetic F2ds were added to make the final concentrations of F2d which were 12.5 μg/mL, 25 μg/mL, and 50 μg/mL, respectively. After 2 h, HUVEC cells were added H2O2 treatments. 10 μL of MTS solution was added to each well and placed in a CO2 incubator for 30 min. The absorbance was measured at 492 nm by using an enzyme-linked immunoassay analyzer. According to the OD value, cell viabilities of different peptides were assessed. [file 2637577.f2.pdf]
